# Experience and severity of menopause symptoms and effects on health-seeking behaviours: a cross-sectional online survey of community dwelling adults in the United Kingdom

**DOI:** 10.1186/s12905-023-02506-w

**Published:** 2023-07-14

**Authors:** David Roy Huang, Abigail Goodship, Iman Webber, Aos Alaa, Eva Riboli Sasco, Benedict Hayhoe, Austen El-Osta

**Affiliations:** Self-Care Academic Research Unit (SCARU), Department of Primary Care & Public Health, Imperial College, London, W6 8RF UK

**Keywords:** Menopause, Patient acceptance of health care, Delivery of health care, Group consultation, Shared medical appointment, Self-care

## Abstract

**Background:**

Almost all women will experience menopause, and the symptoms can have a severely detrimental impact on their quality of life. However, there is limited research exploring health-seeking behaviours and alternative service design or consultation formats. Group consultations have been successfully deployed in perinatal and diabetic care, improving accessibility and outcomes. This cross-sectional online survey was conducted to explore women’s personal experiences of menopause, including perspectives on group consultations.

**Methods:**

An online survey investigated the experiences of individuals at all stages of menopause and their receptiveness towards group consultations for menopause. Respondents were categorised by menopause stage according to the STRAW + 10 staging system. Associations between menopause stage, acceptability of group consultations and participant demographics were assessed using logistic regression.

**Results:**

Respondents experienced an average of 10.7 menopausal symptoms, but only 47% of respondents felt they had the knowledge and tools to manage their symptoms. Advice on menopause was sought from a healthcare professional (HCP) by 61% of respondents, the largest trigger for this being severity of symptoms and the main barrier for this was the perception that menopause wasn’t a valid enough reason to seek help. Of the respondents seeking advice from HCPs, 32% were prescribed transdermal HRT, 29% received oral HRT, 19% were offered antidepressants, 18% received local oestrogen and 6% were prescribed testosterone. Over three quarters (77%) of respondents indicated that they would join a group consultation for menopause and would be comfortable sharing their experiences with others (75%). Logistic regression indicated premenopausal respondents were 2.84 times more likely than postmenopausal women to be interested in a group consultation where they can meet or learn from others’ experiences.

**Conclusions:**

This study highlighted a strong willingness of women aged 35–70 to participate in group consultations for menopause, with motivation being strongest amongst premenopausal women. Low awareness of self-management and lifestyle interventions to manage the symptoms of menopause highlight the need for greater outreach, research and interventions to build knowledge and confidence in the general population at scale. Future studies should focus on investigating the effectiveness and economic impact of menopause group consultations and the lived experience of individuals participating in group consultations.

**Supplementary Information:**

The online version contains supplementary material available at 10.1186/s12905-023-02506-w.

## Introduction

Menopause is defined as the permanent cessation of menstruation, resulting from a decline in ovarian follicular activity [1]. It is preceded by the menopausal transition, a period of menstrual cycle irregularity, which usually begins in the mid 40s, and is followed by the postmenopausal period [2]. By 2025, the number of postmenopausal women worldwide is expected to exceed 1 billion [3]. Despite the ubiquity of menopause, women in the menopausal transition report considerably lower levels of health-related quality of life and higher levels of work impairment [4]. Vasomotor menopausal symptoms are often considered the cardinal symptoms of menopause, and over three quarters of women describe their menopause symptoms as moderately or extremely problematic at work, while 44% have taken undisclosed sickness absence due to their symptoms [5]. An American study found that $27.6 m was lost in work productivity in women with untreated vasomotor symptoms [6]. A study by the UK Department for Work and Pensions estimated that if 600,000 more post-menopausal women continued to work full or part-time, this would add £20bn and £9bn to GDP respectively [7].

The Women’s Health Strategy survey conducted by the Department of Health and Social care found that only 9% of respondents felt that they had enough information on menopause, and considered menopause to be one of top health conditions causing them the highest concern [8]. Hormone replacement therapy (HRT) is the most effective treatment option for menopausal symptoms, yet it is widely under-prescribed by healthcare professionals (HCP). Menopause should also ideally be managed holistically, using positive health behaviours in combination with hormonal or non-hormonal treatments. However, approximately two thirds of menopausal women are unable to access adequate care for their symptoms highlighting the demand for more accessible, up-to-date information around menopause [9]. Awareness of menopause and positive attitudes can influence the severity of symptoms and quality of life. Psychological aspects such as perceived control and self-compassion are more strongly linked to wellbeing than physiological symptoms such as the frequency of hot flushes experienced [10].

Surveys from the Royal College of Obstetricians and Gynaecologists demonstrate 58% percent of women are unable to access menopause services locally, suggesting there is an urgent need to increase access [11]. Primary care providers are responsible for managing the majority of the NHS’ caseload, yet only half of GPs receive menopause training [12]. Secondary care referrals associated with short-term symptom management and long-term conditions are expected to increase, including uptake of HRT, with considerable pressure on scarce NHS resources [13]. One approach to help address these problems may be the use of group consultations [14], also known as shared medical appointments [15].

The use of group consultations has seen increasing popularity in the UK, USA, Australia and India, and especially since the advent of the COVID-19 pandemic where healthcare resources have become increasingly strained. Group consultations have been successfully used to manage an increasing variety of conditions, including diabetes [16] and post-op orthopaedic care [17], leading to improved patient outcomes and satisfaction rates in that same order [18]. Various models of group consultations have been developed that improve access and continuity of care including antenatal group care [19]. Perinatal care offers the most compelling evidence for group healthcare as demonstrated by the popularity of National Childbirth Trust courses in the UK, and the American College of Obstetricians and Gynaecologists’ joint opinion that patients have better prenatal knowledge, initiate breastfeeding more often and are more satisfied with their care [20]. Group consultations can improve patient outcomes relative to individualised patient care and are not usually associated with adverse outcomes [21]. They also offer a special opportunity to deliver more effective care at lower cost. Realising group consultations can bring 300–400% efficiency gains over traditional care models, and the NHS is actively investigating how best to embed group consultations into routine practice [22]. This may be especially beneficial during menopause when timely access to knowledgeable HCPs and quality assured health literacy information is difficult to find and remains inaccessible to many.

Only 54% of women seek medical input for menopausal symptoms, despite > 80% experiencing some combination of symptoms associated with oestrogenic deficiency [23]. European surveys have explored the perceptions and experiences of menopausal women illustrating high levels of understanding and awareness [24, 25], accompanied by a significant negative impact on the psychological and sexual well-being of the individual [26, 27]. However, few studies have investigated the influencing factors that trigger women to seek medical care. The aim of this study was to investigate the prevailing needs and motivations of women when seeking health information around menopause, and to establish their willingness to participate in group-based healthcare, education, and support.

## Methods

### Study design

We conducted a cross-sectional online survey of English-speaking respondents between the ages of 35 and 70 living in the United Kingdom. A structured search was carried out in Pubmed prior to deployment of our survey. Search terms included: “menopause experience”, “perceptions of care,” “patient acceptance”, “delivery of menopause care”, “group consultation”, “shared medical appointment”, and “self-care.” There were multiple references.

The link to the electronic survey was published and made available on the Imperial College Qualtrics platform between 18 November 2020 and 19 March 2021 (4 months). The survey was open and could be accessed by anyone with a link. Survey participants were recruited through affiliates mailing lists, such as partnering organisations and community groups, menopause specialist clinics, and gyms, and online media channels, such as Facebook and Instagram. Potentially eligible participants received an invitation email from the study team. The researchers’ personal and professional networks were also mobilized to respond and further disseminate the eSurvey among potentially eligible participants. The final sample is a convenience sample.

The Participant Information Sheet (PIS) included information regarding the study’s aims, the protection of participants’ personal data, their right to withdraw from the study at any time, which data were stored, where and for how long, who the investigator was, the purpose of the study and survey length. Participants were informed that this was a voluntary survey without any monetary incentives but offering the possibility to access the findings at a later stage whilst underlining the potential collective benefits of taking part in terms of shaping our understanding of what the general population thinks about menopause care and how we can design better services in future. Participants were also given the opportunity to provide their contact details on the occasion that they were interested to be interviewed. The data collected were stored on the Imperial College London secure database and only members of the study team could access the eSurvey results.

The survey adopted adaptive questioning and comprised a total of 28 questions. It was accessible using a personal computer or smartphone. Participants could review their answers before submitting them. Questions regarding demographic characteristics of the users included information on assigned sex at birth, gender, age, ethnicity, educational level, marital status, the name of town or city they lived in and employment status. If participants did not select ‘female’ as their assigned sex at birth, they were automatically excluded. Rather than using the general term of "woman", the assigned sex at birth allows a greater inclusivity since trans men, nonbinary and other gender nonconforming individuals can experience menopause. All data collected through the survey were anonymised and not personally identifiable. The online survey was piloted with a small group of individuals to ensure technical functionality and usability before being published.

Menopausal phase was assessed through questions regarding menopausal symptoms and frequency of or date of last menstrual period. Respondents were categorised into premenopause, perimenopause and postmenopause according to the STRAW + 10 Staging System [28, 29]. The STRAW system categorises women into menopausal stages based on when they had their last period and the variability of their menstrual cycle, as menopause is clinically defined as having occurred 12 months after the final menstrual period. Respondents who indicated they had surgically induced menopause were categorised as surgical menopause.

To investigate menopausal symptoms experienced and the symptoms that acted as triggers for seeking help, the Menopause-Specific Quality Of Life (MENQOL) questionnaire was adapted into a list of 18 symptoms [30, 31]. Self-care behaviours were investigated using a series of questions regarding the respondent's preferred sources of menopause information and advice, personal triggers and barriers for seeking advice and experiences with HCPs. The demand for group consultations was evaluated using a number of statements where participants were asked to indicate level of agreement. The Checklist for Reporting Results of Internet E-Surveys (CHERRIES) [32] was used to guide the development and reporting of the eSurvey.

### Statistical analysis

Respondent characteristics were described using frequencies and percentages. Survey responses to the statement: “I would join a group consultation if that meant I could get personal medical advice from a menopause specialist” were used in statistical analysis and logistic regression models. Associations between menopause stage, acceptability of group consultations and participant demographics were assessed using logistic regression models. All analyses were performed using Stata 15 statistical software (StataCorp). A *p* value 0.05 was considered statistically significant.

### Ethics

The study received a favourable opinion from Imperial College Research Ethics Committee (ICREC #20IC6389). Participants consented to take part in the survey.

### Patient and public involvement

No patients were involved as research participants. The study protocol and online survey were developed in collaboration with Bia Health Ltd, which included input from PPI group and lay members. Preliminary surveys were conducted with approximately 200 women to gauge their attitudes towards group healthcare and experiences of menopause care. The Survey was reviewed by eight menopausal women as part of beta testing on usability and wording of questions.

## Results

### Demographic profile of respondents

The electronic survey captured data from 1547 respondents. We recorded a total of 594 excluded due to duplicate entries (*n* = 7), missing ethnicity (*n* = 498) or missing age (*n* = 235), leaving a sample size of 953.

The majority (68.0%) of respondents were aged between 46 and 55 years (27.7% were 46–50 years and 40.3% were 51–55 years; Table 1). All respondents confirmed their assigned sex at birth was female and 99% identified their gender as female. The remaining 1% identified as "other" including non-binary and agender. In view of this, and for reasons of pragmatism, we opted for the use of the term "women" in this article but remain aware that menopause does not solely concern cisgender women. 88.5% of respondents identified as white ethnic background, and 5.7% identified as Black, Asian, British Black or British Asian. Whereas 66.0% were educated to university degree level or higher, 20.8% were educated to A-Levels, 81.0% were employed, 6.5% were retired and 81.6% were married or in a domestic relationship (Table 1).

**Table 1:** Participant characteristics

	**Pre-menopausal** **(*n* = 27)**		**Peri-menopausal** **(*n* = 390)**		**Menopausal** **(*n* = 410)**		**Post-menopausal** **(*n* = 102)**		**Surgical menopause** **(*n* = 35)**		***P*-value**
**Age,** mean (SD)	44.3	(5.6)	49.5	(4.0)	54.5	(4.2)	55.6	(5.6)	52.7	(5.4)	** < 0.001**
< 39	3	(37.5)	4	(50.0)	0	(0.0)	1	(12.5)	0	(0.0)	
40–45	9	13.9)	46	(70.8)	6	(9.2)	2	(3.1)	2	(3.1)	
46–50	12	(4.6)	173	(65.5)	58	(22.0)	10	(3.8)	11	(4.2)	
51–55	2	(0.4)	163	(28.4)	320	(55.8)	71	(12.4)	18	(3.1)	
56–60	0	(0.0)	1	(2.1)	26	(54.2)	17	(35.4)	4	(8.3)	
**Ethnicity, n (%)**											**0.01**
White	21	(2.5)	345	(40.9)	360	(42.7)	87	(10.3)	31	(3.7)	
Mixed multiple	2	(8.7)	12	(52.2)	6	(26.1)	2	(8.7)	1	(4.4)	
White & black Caribbean	0	(0.0)	9	(33.3)	12	(44.4)	5	(18.5)	1	(3.7)	
Asia/ Asian-British	3	(15)	7	(35.0)	8	(40.0)	2	(10.0)	0	(0.0)	
British Black / African	0	(0.0)	1	(14.3)	4	(57.1)	0	(0.0)	2	(28.6)	
Other	1	(2.3)	16	(37.2)	20	(46.5)	6	(14.0)	0	(0.0)	
**Education, n (%)**											**0.49**
Secondary school	2	(2.4)	25	(29.8)	42	(50.0)	10	(11.9)	5	(6.0)	
A-Levels / College	4	(2.0)	72	(36.4)	93	(47.0)	22	(11.1)	7	(3.5)	
University Degree or higher	20	(3.2)	274	(43.4)	250	(39.6)	67	(10.6)	20	(3.2)	
Other	1	(2.2)	18	(40.0)	21	(46.7)	3	(6.7)	2	(4.4)	
**Employment, n (%)**											**0.05**
Employed full time	11	(3.0)	163	(44.4)	144	(39.2)	39	(10.6)	10	(2.7)	
Employed part-time	4	(1.7)	100	(42.6)	101	(43.0)	22	(9.4)	8	(3.4)	
Self-employed	10	(5.6)	70	(39.3)	74	(41.6)	20	(11.2)	4	(2.2)	
Furloughed	1	(4.8)	8	(38.1)	9	(42.9)	1	(4.8)	2	(9.5)	
Retired	0	(0.0)	16	(25.4)	34	(52.0)	9	(14.3)	4	(6.3)	
Unemployed	1	(1.8)	22	(38.6)	30	(52.6)	2	(3.5)	2	(3.5)	
Unable to work	0	(0.0)	11	(28.9)	17	(44.7)	6	(15.8)	4	(10.5)	
**Marital status**											**0.07**
Married	17	(2.6)	257	(39.8)	290	(44.9)	60	(9.3)	22	(3.4)	
In a domestic relationship	6	(4.3)	58	(41.1)	56	(39.7)	16	(11.3)	5	(3.5)	
Never married	1	(1.5)	32	(48.5)	20	(30.3)	10	(15.2)	3	(4.5)	
Divorced	2	(2.6)	32	(41.0)	33	(42.3)	10	(12.8)	1	(1.3)	
Widowed	0	(0.0)	3	(37.5)	2	(25.0)	3	(37.5)	0	(0.0)	
Other	1	(4.3)	7	(30.4)	8	(34.8)	3	(13.0)	4	(17.4)	
**What triggered you to seek professional advice for your menopause?**											
Severity of symptoms	3	(0.7)	181	(39.3)	214	(46.4)	41	(8.9)	22	(4.8)	** < 0.001**
Concerns over no longer being able to conceive	1	(25.0)	2	(50.0)	0	(0.0)	1	(25.0)	0	(0.0)	**0.04**
To understand whether HRT is suitable	0	(0.0)	87	(34.1)	128	(50.2)	29	(11.4)	11	(4.3)	**0.001**
Concerns over long term health	1	(0.6)	57	(34.5)	76	(46.1)	16	(9.7)	15	(9.1)	** < 0.001**
Recommendation from a friend	1	(2.3)	20	(46.5)	14	(32.6)	6	(14.0)	2	(4.7)	**0.72**
Recommendation from a sibling	0	(0.0)	6	(54.5)	5	(45.5)	0	(0.0)	0	(0.0)	**0.65**
Recommendation from your children	0	(0.0)	2	(50.0)	2	(50.0)	0	(0.0)	0	(0.0)	**0.94**
Recommendation from partner/spouse	0	(0.0)	15	(44.1)	16	(47.1)	2	(5.9)	1	(2.9)	**0.73**
Online research or articles	2	(2.9)	27	(38.6)	29	(41.4)	7	(10.0)	5	(7.1)	**0.61**
Social media	0	(0.0)	11	(45.8)	10	(41.7)	1	(4.2)	2	(8.3)	**0.51**
Other	0	(0.0)	15	(24.6)	31	(50.8)	10	(16.4)	5	(8.2)	**0.01**

### Symptoms experienced by respondents

The mean number of menopausal symptoms experienced by respondents was 10.7. The most common menopausal symptom (81.8%) was sleep disturbances (including difficulty falling asleep, staying asleep or early waking). This was closely followed by hot flushes or night sweats (80.7%) and forgetfulness or memory problems (75.6%). Seventy three percent of respondents experienced symptoms of incontinence, Psychological symptoms were prevalent (72.4%) with low or depressed mood (66.6%) being most common, followed by anxiety or panic attacks (59.7%). 68.3% reported a lower sex drive, whereas 42% of all respondents experienced dry vagina or painful sex. Dry skin, broken hair and nails affected 54.7% of the respondents. Migraines, dizziness, and dry mouth or eyes were the most common ‘Other’ symptoms, experienced by 7% of respondents.

### Sources of menopause advice

Nearly half (46.9%) of respondents felt that they had the tools and understanding to manage their symptoms. Around two thirds (61%) sought help from HCP or doctor. Of those, the vast majority (93.3%) saw an NHS GP or gynaecologist, with very few seeing a psychologist (5.2%), women’s health physiotherapist (4.1%), nutritionist (3.8%) or dietician (1.4%). The largest trigger for seeking advice was severity of symptoms (78.7%). The most common symptom to prompt respondents to seek help from HCP was hot flushes or night sweats (31.3%). Whilst incontinence was experienced by three quarters of participants, incontinence symptoms were amongst the least likely to prompt respondents to seek help (8.1%) (Table S1).

Forty four percent of respondents who saw HCPs were prompted to do this to understand whether HRT was a suitable option. For 71.1% of women, the support provided by their HCP was prescription medication. Transdermal HRT was prescribed to 31.8%, oral HRT to 29.3% and local oestrogen to 18.1%. Only 6.2% of respondents who saw a doctor were prescribed testosterone. Notably, 19.3% of respondents were prescribed SSRIs or SNRIs despite over 14% of this cohort not reporting any vasomotor or psychological symptoms. Lifestyle changes were proposed to 22.9% of those who saw HCPs, either as standalone (7.6%) or in combination with prescribed medication (15.3%).

Excluding HCPs, the most popular sources of advice or information about menopause were health websites (55.4%) and friends (45.1%). However, of those respondents who visited HCP, only 11.5% were prompted to do so by online research or articles and 7.2% by friends. Only 5.2% of women sought advice from pharmacists. The remaining 43.5% of participants who did not seek advice from HCP reported that their symptoms were not severe, or they could manage or cope on their own. One hundred and ninety-five respondents (52.4%) of those who did not seek advice from HCP felt that menopause was not a valid enough reason to be seeking support (i.e. they did not think it menopause was an appropriate reason to get medical advice and/or they did not think HCP could help them and/or that the symptoms they were experiencing warranted HCP time and support). Eighteen respondents (1.8%) described reasons that would fit into the category of not trusting HCP to help them—either due to a fear of misdiagnosis, not being taken seriously by HCPs, or the perception that HCPs lacked specialist menopause knowledge and training.

### Lifestyle management of symptoms

Seventy percent of respondents indicated that they had implemented lifestyle changes to help them self-care for their menopause symptoms; 37.8% reduced their alcohol intake, 35.6% started a new exercise regime, 27.4% reduced caffeine intake, 6.7% started cognitive behavioural therapy and 4.2% stopped smoking. Of those respondents, 82% indicated that these lifestyle changes were moderately effective for symptom relief. Less than half (40.5%) of respondents identified taking supplements such as black cohosh, evening primrose oil or magnesium to help them manage, with 75% of those respondents indicating they had some effectiveness for symptom relief.

Two hundred and eighty four respondents (29.8%) made no behavioural or lifestyle changes. 9.9% of respondents reported making ‘Other’ lifestyle changes. The most common changes were reducing work stress or changing jobs (39.1%) and specific dietary changes, such as removing dairy or wheat from their diet. Of those that made lifestyle changes, 82% report the changes having some effect, with the majority (71.6%) only reporting a slight or moderate effect.

### Acceptability of group consultations

Overall, 77% of participants agreed that they would join a group consultation if it meant that they could get personal medical advice from a menopause specialist. Three quarters (75%) of study participants agreed that they would be comfortable sharing their menopause experiences in a confidential group setting with others. The majority (80%) of premenopausal individuals agreed they would join a group consultation or sessions where they could meet or hear from others’ lived experiences, compared to 63%, 59% and 70% of perimenopausal, postmenopausal and women experiencing surgical menopause, respectively (Table S1).

### Univariable and multivariable association of menopause status with acceptability of group consultations

Univariable logistic regression showed that premenopausal women were 2.84 times more likely than postmenopausal women to be interested in participating in group consultation (Table 2). The association remained significant in the multivariable model after adjusting for ethnicity, education, marital status and employment (Table 2).

**Table 2:** Association of menopause status with acceptability of group consultations

	**Univariable**			**Multivariable**^a^		
**Stage**	**OR**	**95% CI**	***P*-value**	**aOR**	**95% CI**	***P*-value**
Post-menopause	Ref			Ref		
Pre-menopause	2.84	(1.05, 7.69)	0.04	2.79	(1.02, 7.67)	0.05
Peri-menopause	1.21	(0.92, 1.59)	0.17	1.16	(0.88, 1.53)	0.3
Surgical menopause	1.63	(0.76, 3.50)	0.21	1.54	(0.70, 3.42)	0.29

^a^Model adjusted for ethnicity, education, marital status & employment

## Discussion

### Summary of key findings

To our knowledge, this is the first study to investigate the feasibility and acceptability of group consultations for menopause. We analysed data collected over a 4-month period from 953 respondents assigned female at birth, living in the UK aged 35–70 years, across all stages of menopause. Our findings suggest that group consultations would be widely accepted among menopausal populations as the majority (76%) of participants indicated that they would join a group consultation for this purpose.

Compared to other women, pre-menopausal women specifically were most likely to be interested in a menopause group consultation where they could meet and learn about other women’s experiences. The proportion of women interested in menopause group consultations reported in this study exceeded the proportion of pregnant women who were interested in participating in group antenatal care in a similar quantitative survey in 2015 [33]. The prevalence of menopause symptoms among women aged 35–70 reported in our study is broadly consistent with previous literature [34]. As reported here and elsewhere, approximately 8 out of 10 women reported hot flushes and night sweats, which are considered the hallmark indicators of menopause [35]. A similar proportion of respondents suffered from sleep disturbances, although previous studies have indicated lower levels of sleep disturbances in midlife women, ranging between 40 and 56% [36, 37]. The reason for this disparity is difficult to determine because the cause of each woman’s sleep disturbance may be multifactorial, and sleep issues were likely exacerbated by changes in lifestyle and routines during the COVID-19 pandemic [38].

The percentage of women who were prescribed HRT is consistent with the limited literature available [39, 40]; it is interesting to note that our study found that the same proportion of women were prescribed oral HRT and transdermal HRT. This is despite data indicating that the overall risk–benefit profile of transdermal HRT makes it a more attractive option for the majority of menopausal individuals and suggests that more work needs to be done in promoting the use of transdermal HRT [41].

Testosterone can demonstrably improve sexual function and is recommended by the National Institute for Health and Care Excellence (NICE) guidelines for menopausal women with low libido where HRT alone is ineffective [42, 43]. However, our data suggests that testosterone is largely underutilised since prescriptions were only given to 6% of those who saw a healthcare professional, despite 68.3% of participants in our study admitting experiencing symptoms of low libido. There are currently no available licensed testosterone preparations for women in the UK, which could explain the low levels of prescription.

One of the top sources of advice reported here is the use of health websites, suggesting that over the past decade, the popularity of the internet as an advice source for menopause has skyrocketed.

### Need for greater awareness

Group consultations in other areas including diabetes and antenatal care have been largely successful [19, 44]. The findings of our study highlight that the demand for menopause group consultations seems equally as high, if not higher, than in these other healthcare areas.

Over three-quarters of participants indicated that they would join a group consultation if it meant they could meet others or hear others’ experiences of menopause. This suggests that although menopause involves many intimate areas of health, women would enjoy the integrated community aspect of group consultations, although around half of participants had doubts whether group consultations would be as good as a one-to-one consultation. Growing evidence suggests that outcomes from group consultations are the same, or sometimes better than one-to-one consultations, and some even preferred groups given the relationship-building aspect in addition to extended time with the specialist [22]. Here there was a clear mismatch between the perceptions of group consultations and initial preferences, which indicated the need for improved education surrounding the benefits of group consultations. It is interesting to consider why women earlier on in their menopausal journey were more receptive and whether this pertains to their relative lack of experience with menopause or better knowledge of the benefits of group consultations.

### Implications for policy and practice

Further implications for menopause healthcare policy and practice can be identified from our findings around patient experiences and health-seeking behaviours. The mean number of symptoms reported by respondents was 10.7, indicating the diversity of symptoms experienced by menopausal women. This suggests that clinicians need to be more aware of the broad range of self-care strategies available to support individuals. For example, for menopausal patients suffering from insomnia, CBT-i could be recommended as it is largely considered to be the first line of treatment for insomnia [45]. Another key point regarding symptoms is despite the high percentage of women experiencing urogenital symptoms, only a small percentage of those are prompted to seek help as a result of their symptoms. This suggests that women are either unaware that these are treatable menopause symptoms or don’t feel comfortable seeking help. A longer dwell time with clinicians within a group consultation setting could not only help destigmatise but would also allow for an exploration of front-line therapy options, including vaginal oestrogen or non-hormonal management strategies such as lifestyle changes, vaginal moisturiser or lubricants [46].

In the UK, there are 449 million visits to community pharmacies annually, and evidence suggests that integrating pharmacists into primary care could help reduce GP workload. Our data also indicated that pharmacists are an underutilised resource and would be ideally placed to offer quality assured menopause advice [47]. However, as over half of participants did not seek advice because they did not think menopause was a valid enough reason to schedule an appointment with a HCP, this indicates that as a society we need to change the narrative to let women know that their experiences are valid, and that it is important to seek help for any symptom that adversely affects their lived experience or quality of life. HCPs also need to change attitudes and perceptions towards menopause and be proactive in providing help to those that need it. Worryingly, a number of participants demonstrated a lack of trust in their clinicians to be able to provide them with the support they needed, which could relate to the widespread lack of menopause training for doctors. Group consultations are an opportunity to educate patients and better utilise HCPs that have been trained.

### Study strengths and limitations

To our knowledge, this is the first study to investigate the feasibility and acceptability of group consultations for menopause in the UK.

The principal limitation of this study was that we collected data between November 2020 and March 2021. This time horizon included national lockdowns and so we need to take into consideration that the COVID-19 pandemic may have influenced personal experiences of menopause and that general accessibility to menopause care also is reported to have declined [48]. We also acknowledge that because the survey was conducted and distributed electronically, we may have excluded individuals with limited digital access. Additionally, the demographic profile of study participants largely consisted of university-educated cisgender women, implying that this cross-section may not be representative of the wider UK population experiencing menopause.

### Further research

This study sheds some important light on women’s preferences on what information they would like to receive about menopause and the value of group consultations with input from a prescribing clinician who could streamline access to HRT as needed. Whilst there is limited evidence available on the clinical efficacy of menopause group consultations specifically, the substantial need for further support for women in accessing healthcare advice and treatment relating to the menopause identified in this study, and the evident interest of women in group consultations for this purpose, indicates that group consultations for menopause should now be trialled to determine the extent they are able to improve clinical outcomes in the context of the NHS and real-world healthcare settings. We can use the data collected here to target women who are more likely to be open to group consultations, consequently increasing the potential for self-care and improvements in patient activation measures.

Further studies might also investigate the evolving landscape in the medical management of menopause, including the role of groups in accelerating the dissemination of information and the uptake of novel therapeutic options. Examples include the changing prescribing patterns of hormonal therapies, such as testosterone for libido and fatigue symptoms, or the use of selective oestrogen receptor modulators (SERMs) for vulvovaginal atrophy or overactive bladder symptoms [49, 50]. In addition, SERMs and emerging non-hormonal therapeutics, including neurokinin 3 receptor antagonists [51], have additional utility when managing patients with a history of breast cancer who otherwise have limited options and have shown benefit from group and peer support [52].

## Conclusion

Our study highlights a strong willingness of women aged 35–70 to participate in group consultations for menopause and demonstrates that this motivation is strongest amongst premenopausal women. Our survey also indicated that only a small percentage of women felt that they had the tools and understanding to manage menopause necessitating further research and interventions to build knowledge and confidence in the general population at scale. Using group consultations for first-line menopause healthcare provision should be strongly considered to improve accessibility and meet growing global demand for care.

Future studies should focus on investigating the effectiveness and economic impact of menopause group consultations and the experiences of individuals in those consultations. Within these studies, the effect of group size and composition should be evaluated. Additionally, remote group consultations should be compared to in-person group consultations, as remote consultations would decrease the resources needed and increase accessibility.

## Supplementary information

**Additional file 1:** **TableS1.  **Demographics, health-seekingbehaviours and healthcare experiences. 

## Data Availability

The datasets used and/or analysed during the current study available from the corresponding author on reasonable request.

## References

[1] Mosconi L, Berti V, Dyke J, Schelbaum E, Jett S, Loughlin L (2021). Menopause impacts human brain structure, connectivity, energy metabolism, and amyloid-beta deposition. Sci Rep.

[2] Auro K, Joensuu A, Fischer K, Kettunen J, Salo P, Mattsson H (2014). A metabolic view on menopause and ageing. Nat Commun.

[3] Shifren JL, Gass MLS (2014). The North American menopause society recommendations for clinical care of midlife women. Menopause.

[4] Whiteley J, DiBonaventura M daCosta, Wagner JS, Alvir J, Shah S. The Impact of Menopausal Symptoms on Quality of Life, Productivity, and Economic Outcomes. J Womens Health. 2013 Nov;22(11):983–90. 10.1089/jwh.2012.3719 PMC3820128 24083674

[5] Boag-Munroe DF. Menopause Survey Headline Statistics April 2019. 2018 [cited 2023 Feb 13]; Available from: https://www.polfed.org/media/14789/menopause-survey-2018-derbyshire-constabulary-18-03-19-v10.pdf

[6] Sarrel P, Portman D, Lefebvre P, Lafeuille MH, Grittner AM, Fortier J (2015). Incremental direct and indirect costs of untreated vasomotor symptoms. Menopause.

[7] Cbe DRA. A new vision for older workers: retain, retrain, recruit. Available from: https://assets.publishing.service.gov.uk/government/uploads/system/uploads/attachment_data/file/411420/a-new-vision-for-older-workers.pdf

[8] Secretary of State for Health and Social Care. Women’s Health Strategy for England. 2022.

[9] Marlatt KL, Beyl RA, Redman LM (2018). A qualitative assessment of health behaviors and experiences during menopause: A cross-sectional, observational study. Maturitas.

[10] Brown L, Bryant C, Brown V, Bei B, Judd F (2015). Investigating how menopausal factors and self-compassion shape well-being: An exploratory path analysis. Maturitas.

[11] Better for women - Improving the health and wellbeing of girls and women [Internet]. Royal College of Obstetricians and Gynaecologists; 2019 [cited 2023 Feb 13]. Available from: https://www.rcog.org.uk/media/h3smwohw/better-for-women-full-report.pdf

[12] Newson L, Mair R. Results From The BJFM Menopause Survey [Internet]. Pavilion Health Today - Family Medicine; 2018. Available from: https://www.pavilionhealthtoday.com/fm/results-from-the-bjfm-menopause-survey/

[13] Gambacciani M, Cagnacci A (2022). HRT in a new light: Thanks UK. Maturitas.

[14] Gibson H, Huang D, Hayhoe BWJ, El-Osta A. A Virtual Group Consultation Model of Care for Menopause Treatment. NEJM Catal [Internet]. 2022 May 18 [cited 2023 Feb 17];3(6). Available from:10.1056/CAT.22.0041

[15] Hayhoe B, Verma A, Kumar S (2017). Shared medical appointments. BMJ.

[16] Sadikot SM, Das AK, Wilding J, Siyan A, Zargar AH, Saboo B (2017). Consensus recommendations on exploring effective solutions for the rising cost of diabetes. Diabetes Metab Syndr Clin Res Rev.

[17] Powell RL, Biernacki PJ (2019). Shared Medical Appointments in Preoperative Joint Replacement: Assessing Patient and Healthcare Member Satisfaction. J Healthc Qual.

[18] Edelman D, Gierisch JM, McDuffie JR, Oddone E, Williams JW (2015). Shared medical appointments for patients with diabetes mellitus: a systematic review. J Gen Intern Med.

[19] Cunningham SD, Lewis JB, Thomas JL, Grilo SA, Ickovics JR (2017). Expect With Me: development and evaluation design for an innovative model of group prenatal care to improve perinatal outcomes. BMC Pregnancy Childbirth.

[20] ACOG Committee Opinion No (2018). 731 Summary: Group Prenatal Care. Obstet Gynecol.

[21] Catling CJ, Medley N, Foureur M, Ryan C, Leap N, Teate A, et al. Group versus conventional antenatal care for women. Cochrane Pregnancy and Childbirth Group, editor. Cochrane Database Syst Rev [Internet]. 2015 Feb 4 [cited 2023 Feb 12];2017(1). Available from: 10.1002/14651858.CD007622.pub3 10.1002/14651858.CD007622.pub3 PMC6465187 25922865

[22] Jones T, Darzi A, Egger G, Ickovics J, Noffsinger E, Ramdas K (2019). Process and Systems: A systems approach to embedding group consultations in the NHS. Future Healthc J.

[23] Constantine GD, Graham S, Clerinx C, Bernick BA, Krassan M, Mirkin S (2016). Behaviours and attitudes influencing treatment decisions for menopausal symptoms in five European countries. Post Reprod Health.

[24] Vaccaro CM, Capozzi A, Ettore G, Bernorio R, Cagnacci A, Gambacciani M (2021). What women think about menopause: An Italian survey. Maturitas.

[25] Depypere H, Pintiaux A, Desreux J, Hendrickx M, Neven P, Marchowicz E (2016). Coping with menopausal symptoms: An internet survey of Belgian postmenopausal women. Maturitas.

[26] Hoga L, Rodolpho J, Gonçalves B, Quirino B (2015). Women’s experience of menopause: a systematic review of qualitative evidence. JBI Database Syst Rev Implement Rep.

[27] Nappi RE, Nijland EA (2008). Women’s perception of sexuality around the menopause: outcomes of a European telephone survey. Eur J Obstet Gynecol Reprod Biol.

[28] Soules MR, Sherman S, Parrott E, Rebar R, Santoro N, Utian W (2001). Stages of Reproductive Aging Workshop (STRAW). J Womens Health Gend Based Med.

[29] Harlow SD, Gass M, Hall JE, Lobo R, Maki P, Rebar RW (2012). Executive summary of the Stages of Reproductive Aging Workshop + 10: addressing the unfinished agenda of staging reproductive aging. Menopause.

[30] Hilditch JR, Lewis J, Peter A, van Maris B, Ross A, Franssen E (1996). A menopause-specific quality of life questionnaire: development and psychometric properties. Maturitas.

[31] Sydora BC, Fast H, Campbell S, Yuksel N, Lewis JE, Ross S (2016). Use of the Menopause-Specific Quality of Life (MENQOL) questionnaire in research and clinical practice: a comprehensive scoping review. Menopause.

[32] Eysenbach G (2004). Improving the Quality of Web Surveys: The Checklist for Reporting Results of Internet E-Surveys (CHERRIES). J Med Internet Res.

[33] McDonald SD, Sword W, Eryuzlu LN, Neupane B, Beyene J, Biringer AB (2016). Why Are Half of Women Interested in Participating in Group Prenatal Care?. Matern Child Health J.

[34] Monteleone P, Mascagni G, Giannini A, Genazzani AR, Simoncini T (2018). Symptoms of menopause — global prevalence, physiology and implications. Nat Rev Endocrinol.

[35] Bansal R, Aggarwal N (2019). Menopausal hot flashes: A concise review. J -Life Health.

[36] Kravitz HM, Ganz PA, Bromberger J, Powell LH, Sutton-Tyrrell K, Meyer PM. Sleep difficulty in women at midlife: a community survey of sleep and the menopausal transition *: Menopause. 2003;10(1):19–28. 10.1097/00042192-200310010-00005 12544673

[37] Owens JF, Matthews KA (1998). Sleep disturbance in healthy middle-aged women. Maturitas.

[38] Monterrosa-Castro Á, Monterrosa-Blanco A (2021). Prevalencia de problemas de sueño en mujeres climatéricas colombianas durante la pandemia COVID-19. Rev Colomb Obstet Ginecol.

[39] Maclennan AH, Wilson DH, Taylor AW (1998). Hormone replacement therapy: Prevalence, compliance and the ‘healthy women’ notion. Climacteric.

[40] Cumming GP, Currie H, Morris E, Moncur R, Lee AJ (2015). The need to do better – Are we still letting our patients down and at what cost?. Post Reprod Health.

[41] Liu B (2013). Is transdermal menopausal hormone therapy a safer option than oral therapy?. Can Med Assoc J.

[42] Lumsden MA. Menopause: diagnosis and management. Manchester: National Institute for Health and Clinical Excellence; 2015.

[43] Scott A, Newson L (2020). Should we be prescribing testosterone to perimenopausal and menopausal women? A guide to prescribing testosterone for women in primary care. Br J Gen Pract.

[44] Burke RE, Ferrara SA, Fuller AM, Kelderhouse JM, Ferrara LR. The effectiveness of group medical visits on diabetes mellitus type 2 (dm2) specific outcomes in adults: a systematic review: JBI Database Syst Rev Implement Rep. 2011;9(23):833–85. 10.11124/01938924-201109230-00001 27820218

[45] Baglioni C, Altena E, Bjorvatn B, Blom K, Bothelius K, Devoto A, et al. The European Academy for Cognitive Behavioural Therapy for Insomnia: An initiative of the European Insomnia Network to promote implementation and dissemination of treatment. J Sleep Res [Internet]. 2020 Apr [cited 2023 Feb 12];29(2). Available from: 10.1111/jsr.12967 10.1111/jsr.12967 31856367

[46] Singh S, van Herwijnen I, Phillips C (2013). The management of lower urogenital changes in the menopause. Menopause Int.

[47] Hayhoe B, Cespedes JA, Foley K, Majeed A, Ruzangi J, Greenfield G (2019). Impact of integrating pharmacists into primary care teams on health systems indicators: a systematic review. Br J Gen Pract.

[48] Karim F, Araklitis G, Robinson D, Cardozo L (2020). Practice observed in managing gynaecological problems in post-menopausal women during the COVID-19 pandemic. Post Reprod Health.

[49] Di Donato V, Schiavi MC, Iacobelli V, D’oria O, Kontopantelis E, Simoncini T (2019). Ospemifene for the treatment of vulvar and vaginal atrophy: A meta-analysis of randomized trials. Part I Eval Efficacy Matur.

[50] Schiavi MC, Zullo MA, Faiano P, D’Oria O, Prata G, Colagiovanni V (2017). Retrospective analysis in 46 women with vulvovaginal atrophy treated with ospemifene for 12 weeks: improvement in overactive bladder symptoms. Gynecol Endocrinol Off J Int Soc Gynecol Endocrinol.

[51] Prague JK (2021). Neurokinin 3 receptor antagonists – prime time?. Climacteric.

[52] Hu J, Wang X, Guo S, Chen F, Wu Y yu, Ji F jian, et al. Peer support interventions for breast cancer patients: a systematic review. Breast Cancer Res Treat. 2019;174(2):325–41. 10.1007/s10549-018-5033-2 30600413

