# Supplementary material for: Experience and severity of menopause symptoms and effects on health-seeking behaviours: a cross-sectional online survey of community dwelling adults in the United Kingdom
Source: BMC Womens Health. 2023 Jul 14;23:373. doi: 10.1186/s12905-023-02506-w (PMC10347781; doi:10.1186/s12905-023-02506-w)
Supplement: Supplementary file 1 — Additional file 1: TableS1. Demographics, health-seekingbehaviours and healthcare experiences. [file 12905_2023_2506_MOESM1_ESM.docx]

**ESME STUDY - SUPPLEMENTARY DATA**

**Table S1: Demographics, health-seeking behaviours and healthcare experiences**

|  | **Total frequency (n=953)** | **Total frequency as percentage of respondents** |
| --- | --- | --- |
| **Age, mean (SD)** = 52 (5.0) |  |  |
| 35-40 | 9 | 0.9 |
| 41-45 | 59 | 6.2 |
| 46-50 | 264 | 27.7 |
| 51-55 | 384 | 40.3 |
| 56-60 | 190 | 19.9 |
| 61-65 | 37 | 3.9 |
| 66-70 | 10 | 1.0 |
| **Ethnicity** |  |  |
| White | 838 | 87.9 |
| Mixed/Multiple ethnic groups | 20 | 2.1 |
| White and Black Caribbean | 27 | 2.8 |
| Asian/Asian-British | 19 | 2.0 |
| British Black/African/Caribbean | 7 | 0.7 |
| Other | 42 | 4.4 |
| **Education** |  |  |
| Secondary school | 82 | 8.6 |
| A-Levels/College | 197 | 20.8 |
| University Degree or higher | 624 | 65.8 |
| Other | 45 | 4.7 |
| **Employment** |  |  |
| Employed full time | 363 | 38.3 |
| Employed part-time | 231 | 24.3 |
| Self-employed | 177 | 18.7 |
| Furloughed | 21 | 2.2 |
| Retired | 62 | 6.5 |
| Unemployed | 57 | 6.0 |
| Unable to work | 38 | 4.0 |
| **Marital status** |  |  |
| Married | 638 | 67.1 |
| In a domestic relationship | 138 | 14.5 |
| Never married | 66 | 6.9 |
| Divorced | 78 | 8.2 |
| Widowed | 8 | 0.8 |
| Other | 23 | 2.4 |
| **Have you experienced any of the following symptoms?** |  |  |
| Sleep problems including falling asleep, staying asleep or early waking | 780 | 81.8 |
| Hot flushes or night sweats | 769 | 80.7 |
| Forgetfulness or memory problems (brain fog) | 720 | 75.6 |
| Lower sex drive (loss of libido) | 651 | 68.3 |
| Pain in your joints or muscles | 650 | 68.2 |
| Low or depressed mood | 635 | 66.6 |
| Reduced motivation | 607 | 63.7 |
| Irritability | 606 | 63.6 |
| Weight gain | 600 | 63.0 |
| Anxiety or panic attacks | 569 | 59.7 |
| Dry skin, broken hair or nails | 521 | 54.7 |
| Awareness of your heartbeat racing or skipping | 518 | 54.4 |
| Leaking urine when you laugh or cough | 463 | 48.6 |
| Irregular, lighter or heavier periods | 459 | 48.2 |
| Passing urine more frequently (day or night) | 424 | 44.5 |
| Sudden urge to pass urine | 421 | 44.2 |
| Dry vagina or painful sex | 400 | 42.0 |
| Cold flashes | 199 | 20.9 |
| None | 7 | 0.7 |
| Other | 130 | 13.6 |
| **Which symptoms did you see a healthcare professional for, if any?** |  |  |
| Hot flushes or night sweats | 298 | 31.3 |
| Low or depressed mood | 232 | 24.3 |
| Anxiety or panic attacks | 227 | 23.8 |
| Sleep problems including falling asleep, staying asleep or early waking | 202 | 21.2 |
| Pain in your joints or muscles | 175 | 18.4 |
| Forgetfulness or memory problems (brain fog) | 148 | 15.5 |
| Irregular, lighter or heavier periods | 140 | 14.7 |
| Dry vagina or painful sex | 128 | 13.4 |
| Awareness of your heartbeat racing or skipping | 119 | 12.5 |
| Irritability | 118 | 12.4 |
| Lower sex drive (loss of libido) | 90 | 9.4 |
| Reduced motivation | 73 | 7.7 |
| Weight gain | 66 | 6.9 |
| Dry skin, broken hair or nails | 57 | 6.0 |
| Passing urine more frequently (day or night) | 46 | 4.8 |
| Sudden urge to pass urine | 39 | 4.1 |
| Leaking urine when you laugh or cough | 38 | 4.0 |
| Cold flashes | 25 | 2.6 |
| None | 280 | 29.4 |
| Other | 42 | 4.4 |
| **Have you sought menopause advice or information from any of the following?** |  |  |
| Healthcare Professional / Doctor | 581 | 61.0 |
| Health websites (such as NHS, Rock My Menopause, Women's Health Concern or Newson Health) | 528 | 55.4 |
| Friends | 430 | 45.1 |
| News websites (such as the Guardian or BBC) | 325 | 34.1 |
| Online forums (such as Mumsnet) | 270 | 28.3 |
| Healthcare professionals on social media (such as on Instagram) | 193 | 20.3 |
| Magazine websites (such as Women's Health or Hello) | 190 | 19.9 |
| Family | 174 | 18.3 |
| Television or Radio | 126 | 13.2 |
| Blog websites | 120 | 12.6 |
| Pharmacist | 50 | 5.2 |
| Other | 101 | 10.6 |
| **If sought advice from HP: which type of HP did you go to?** |  |  |
| NHS GP or gynaecologist | 542 | 93.3 |
| Private menopause specialist doctor or nurse | 87 | 15.0 |
| NHS menopause specialist doctor or nurse | 86 | 14.8 |
| Psychologist | 30 | 5.2 |
| Women's Health Physiotherapist | 24 | 4.1 |
| Nutritionist | 22 | 3.8 |
| Dietician | 8 | 1.4 |
| Other (please state): | 70 | 12.0 |
| **If sought advice from HP: what triggered you to seek advice?** |  |  |
| Severity of symptoms | 457 | 78.7 |
| To understand whether hormone replacement therapy (HRT) is suitable | 253 | 43.5 |
| Concerns over long term health | 162 | 27.9 |
| Online research or articles | 67 | 11.5 |
| Recommendation from a friend | 42 | 7.2 |
| Recommendation from your partner or spouse | 33 | 5.7 |
| Social media | 20 | 3.4 |
| Recommendation from a sibling | 9 | 1.5 |
| Recommendation from your children | 3 | 0.5 |
| Concerns over no longer being able to conceive | 2 | 0.3 |
| Other (please state): | 60 | 10.3 |
| **If sought advice from HP: what advice did you receive?** |  |  |
| Prescription medication | 413 | 71.1 |
| Lifestyle changes (i.e. diet, exercise) | 133 | 22.9 |
| Referral to another doctor or specialist | 93 | 16 |
| Supplements or non-prescription items | 77 | 13.3 |
| None | 39 | 6.7 |
| Other (please state): | 81 | 13.9 |
| **If sought advice from HP: what medication were you prescribed?** |  |  |
| Transdermal HRT (skin patches, gels or sprays) | 185 | 31.8 |
| Oral Hormone Replacement Therapy (HRT) | 170 | 29.3 |
| Anti-depressants (such as Fluoxetine, Paroxetine or Venlafaxine) | 112 | 19.3 |
| Vaginal HRT (vaginal creams, vaginal tablets or pessaries) | 105 | 18.1 |
| Mirena Coil | 57 | 9.8 |
| Testosterone (such as Androfeme or Testogel) | 36 | 6.2 |
| Propranolol | 15 | 2.6 |
| Clonidine | 12 | 2.1 |
| Gabapentin | 4 | 0.7 |
| Other (please state): | 51 | 8.8 |
| **If didn't seek advice from HP: why not?** |  |  |
| I could manage or cope on my own, my menopause symptoms weren't that severe | 162 | 43.5 |
| I didn't want to waste the healthcare professional's time | 108 | 29 |
| I didn't think they could help me | 94 | 25.3 |
| I didn't think it was an appropriate reason to get medical advice | 88 | 23.7 |
| I want to treat my menopause naturally, without medical advice | 77 | 20.7 |
| I couldn't get an appointment with a knowledgeable healthcare professional | 33 | 8.9 |
| I was embarrassed about my symptoms | 23 | 6.2 |
| I trust my family and friends to give me information and advice | 12 | 3.2 |
| Other (please state): | 65 | 17.5 |
| **Are you taking any non-prescription medicines or supplements to help you manage?** |  |  |
| Yes | 383 | 40.4 |
| No | 564 | 59.6 |
| How effective have they been for symptom relief? |  |  |
| Very effective | 49 | 12.8 |
| Moderately effective | 127 | 33.2 |
| Slightly effective | 114 | 29.8 |
| Not effective at all | 24 | 6.3 |
| I'm not sure | 68 | 17.8 |
| **Have you made any changes to your lifestyle or daily habits to help you prepare?** |  |  |
| Reducing alcohol | 360 | 37.8 |
| Reducing caffeine | 261 | 27.4 |
| Stopping smoking | 40 | 4.2 |
| New exercise regime | 339 | 35.6 |
| New diet regime | 243 | 25.5 |
| Cognitive behavioural therapy | 64 | 6.7 |
| Health, career or life coaching | 48 | 5.0 |
| No change | 284 | 29.8 |
| Other (please specify): | 94 | 9.9 |
| **How effective have these lifestyle changes been for symptom relief?** |  |  |
| Very effective | 68 | 10.4 |
| Moderately effective | 235 | 35.8 |
| Slightly effective | 235 | 35.8 |
| Not effective at all | 66 | 10 |
| I'm not sure | 53 | 8.1 |
| **Do you feel that you currently have the tools and understanding to manage your symptoms** |  |  |
| Definitely yes | 106 | 11.2 |
| Probably yes | 339 | 35.7 |
| Might or might not | 215 | 22.6 |
| Probably not | 238 | 25.1 |
| Definitely not | 52 | 5.5 |
| **If available, which of the following would you be interested in participating in?** |  |  |
| Somewhere I can participate in a live group Q&A on menopause | 217 | 22.8 |
| A group education course where I can learn about menopause | 300 | 31.5 |
| A confidential group where I can talk to other individuals experiencing menopause | 325 | 34.1 |
| A confidential group where I can receive personal medical advice from a menopause specialist | 587 | 61.6 |
| I would not be interested in any of the above | 227 | 23.8 |
| Other type of small group activity (please specify): | 36 | 3.8 |
| **I would join a group consultation or session to meet or hear from others' experiences.** |  |  |
| Strongly disagree | 97 | 10.3 |
| Somewhat disagree | 118 | 12.5 |
| Neither agree nor disagree | 148 | 15.7 |
| Somewhat agree | 344 | 36.5 |
| Strongly agree | 235 | 24.9 |
| **I would be comfortable sharing my menopause experiences in a confidential group setting with others.** |  |  |
| Strongly disagree | 51 | 5.7 |
| Somewhat disagree | 80 | 8.9 |
| Neither agree nor disagree | 92 | 10.2 |
| Somewhat agree | 322 | 35.7 |
| Strongly agree | 357 | 39.6 |
| **I would join a group consultation if that meant I could get personal medical advice from a menopause specialist.** |  |  |
| Strongly disagree | 51 | 5.7 |
| Somewhat disagree | 64 | 7.1 |
| Neither agree nor disagree | 93 | 10.4 |
| Somewhat agree | 279 | 31.1 |
| Strongly agree | 410 | 45.7 |
| **I would join a group consultation if it was less expensive than a 1:1 private consultation with a menopause specialist.** |  |  |
| Strongly disagree | 74 | 8.4 |
| Somewhat disagree | 72 | 8.2 |
| Neither agree nor disagree | 165 | 18.8 |
| Somewhat agree | 243 | 27.6 |
| Strongly agree | 326 | 37 |
| **I wouldn't join a group consultation because it won't be as helpful as a 1:1 consultation.** |  |  |
| Strongly disagree | 160 | 18.5 |
| Somewhat disagree | 252 | 29.2 |
| Neither agree nor disagree | 256 | 29.7 |
| Somewhat agree | 119 | 13.8 |
| Strongly agree | 76 | 8.8 |
| **Menopause status** |  |  |
| Pre-menopause | 25 | 2.6 |
| Perimenopause | 417 | 43.8 |
| Postmenopause | 478 | 50.2 |
| Surgical menopause | 33 | 3.5 |
